# Supplementary material for: Diverse mechanisms of metaeffector activity in an intracellular bacterial pathogen, Legionella pneumophila
Source: Mol Syst Biol. 2016 Dec 16;12(12):893. doi: 10.15252/msb.20167381 (PMC5199130; doi:10.15252/msb.20167381)
Supplement: Supplementary file 1 — Appendix [file MSB-12-893-s001.pdf]

Appendix Figures S1-S9

From: Diverse mechanisms of metaeffector activity in an intracellular bacterial pathogen, *Legionella pneumophila*. (Urbanus, Quaile, *et al*)

Appendix Figure S1 – Automated spot size quantification of two IDTS libraries in *S. cerevisiae.*

Appendix Figure S2 – Suppression of IDTS yeast array strains by query strains.

Appendix Figure S3 – SidP and MavQ supporting data.

Appendix Figure S4 – RavJ has similarities to papain-like cysteine protease family members.

Appendix Figure S5 – Identification of the RavJ domain interacting with LegL1; RavJ-septin complex co-immunoprecipitation.

Appendix Figure S6 – Lpg1148 (LupA) has similarities to ubiquitin or ubiquitin-like protease (UBP) family members.

Appendix Figure S7 – The physical interaction between MavQ and SidP is conserved in *L. dumoffii*.

Appendix Figure S8 – SidP and Lem14 supporting information.

Appendix Figure S9 - Differential transcription of effectors and their antagonists.

Figure S1 - Automated spot size quantification of two IDTS libraries in *S. cerevisiae.*

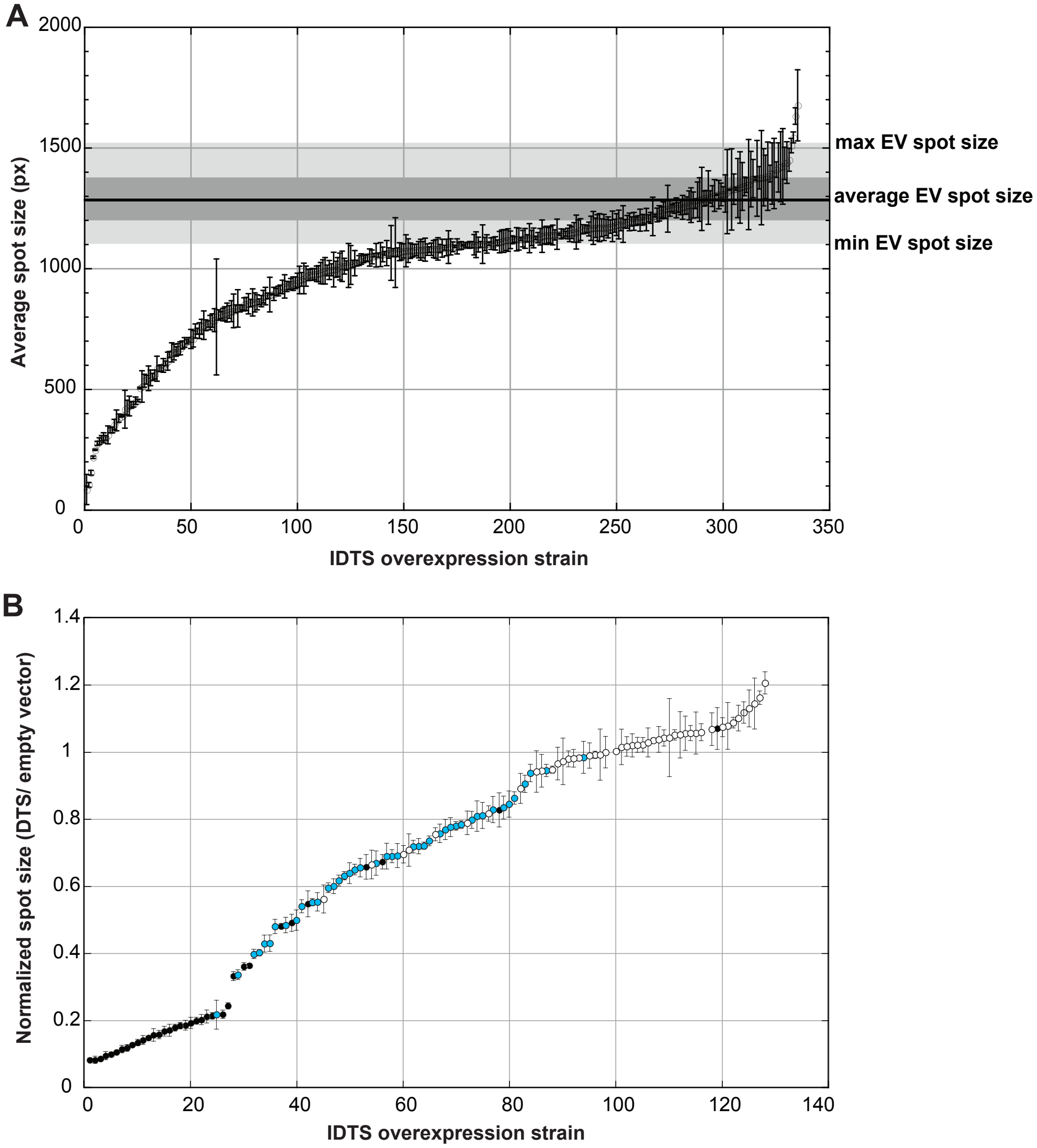

(A) The growth defect for each individual IDTS overexpressing strain in the galactose-inducible BY4741 + pAG423GAL-HA-IDTS library was determined using high-density, automated spot-size measurements. The library was arrayed at 1536-spot density and pinned onto inducing agar plates. After two days of growth, automated image analysis (SGAtools) was used to quantify the spot size of each strain. Error bars represent standard deviation of replicate spots done in quadruplicate. Each strain is plotted in rank order of increasing spot size on the X-axis. The average (line), standard deviation (dark gray box) and the minimum and maximum spot size (light gray box) of 86 empty vector (EV) strain spots are shown. In total 234 IDTS overexpression strains have spot size growth defect, determined as (Average spot size + StdDev) < (Average empty vector control-StdDev). **(B)** A well-characterized yeast expression library (Heidtman, M. *et al* 2009. Cell Microbiol, 11, 230-48, BY4742 + pYES2 NT/A IDTS) in which each strain expresses one of 127 IDTS under the control of a galactose-inducible promoter was grown and quantified as above. The average spot size of each strain is normalized to the empty vector control average and corressponds well to the previous semi-quantitative categorization of the level of yeast toxicity observed for each clone (in Heidtman, M. *et al* 2009. Cell Microbiol, 11, 230-48) indicated as black (++, very toxic), blue (+, moderately toxic), and white (-,non-toxic).

Figure S2 - Suppression of IDTS yeast array strains by query strains.

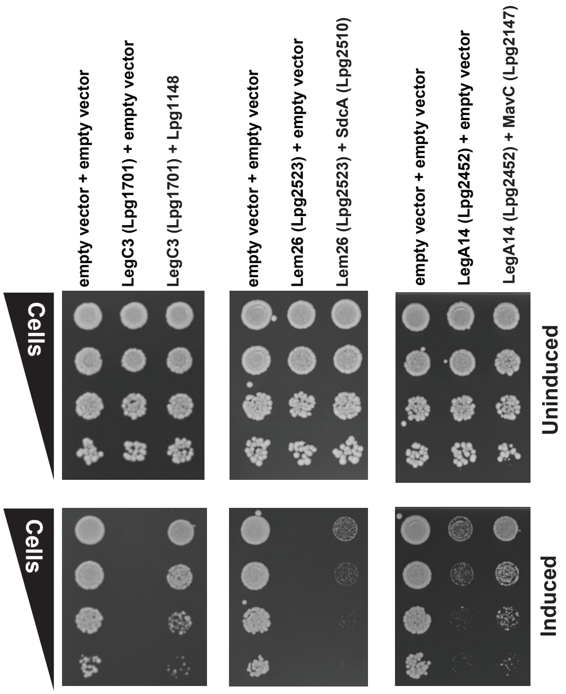

Suppression of yeast growth inhibitory phenotypes in IDTS array strains by IDTS query strains was confirmed by spot dilution assay (see Materials and Methods) under uninduced (2% glucose) and induced (2% galactose) conditions. The spot dilution assay shows suppression of yeast growth inhibition of LegC3, Lem26 and LegA14 (panels left to right) by query strains overexpressing Lpg1148, SdcA and MavC, respectively.

Figure S3 - SidP and MavQ supporting data.

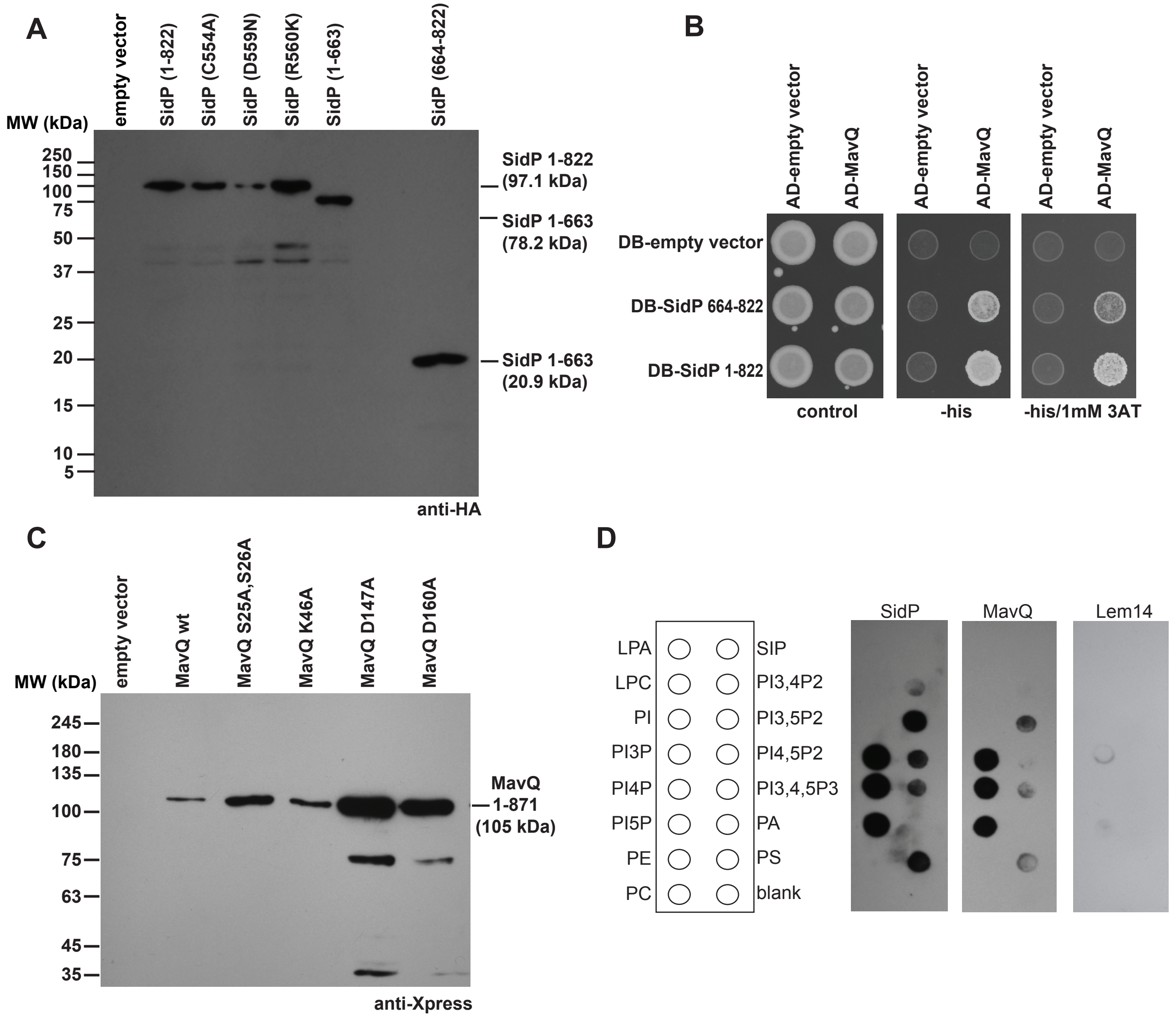

(A) The SidP mutants tested for the ability to alleviate MavQ yeast growth inhibition in a spot dilution assay, are expressed and migrate approximately at their expected molecular weight (including 2.7 kDA for 1X HA and linker sequence) as indicated. Samples of cells induced for 5 hours with galactose were analyzed by SDS-PAGE and Western blot using anti-HA antibodies as described in the Methods. **(B)** The C-terminal domain of SidP interacts with MavQ. MavQ was fused to the gal4-activating domain (AD), and the C-terminal domain of SidP or full-length SidP was fused to the Gal4 DNA-binding domain (DB). Physical interactions between DB and AD drive the expression of the HIS3 gene and are manifested as the ability for a strain to grow under –his and -his/1 mM 3AT conditions. The C-terminal domain of SidP retains the physical interaction with MavQ. **(C)** MavQ mutants in the putative kinase domain tested in a yeast spot dilution assay are expressed and migrate at their expected weight (including or 4.2 kDa for the Xpress tag). Yeast cultures were grown and induced as described in A and analyzed SDS-PAGE and Western blot using anti-Xpress antibodies as described in the Methods. **(D)** MavQ binds to phosphoinositides (PIPs) in a lipid overlay assay. Nitrocellulose membranes on which 100 pmol of various lipids were spotted, were incubated with 5 pmol of purified his6-SBP tagged SidP, MavQ and Lem14 (see Methods). After washing, lipid-bound protein was visualized with anti-His6 and anti-mouse-HRP. Both SidP and MavQ bind to several PIPs and phosphatidyl serine (PS).

Figure S4 - RavJ has similarities to papain-like cysteine protease family members.

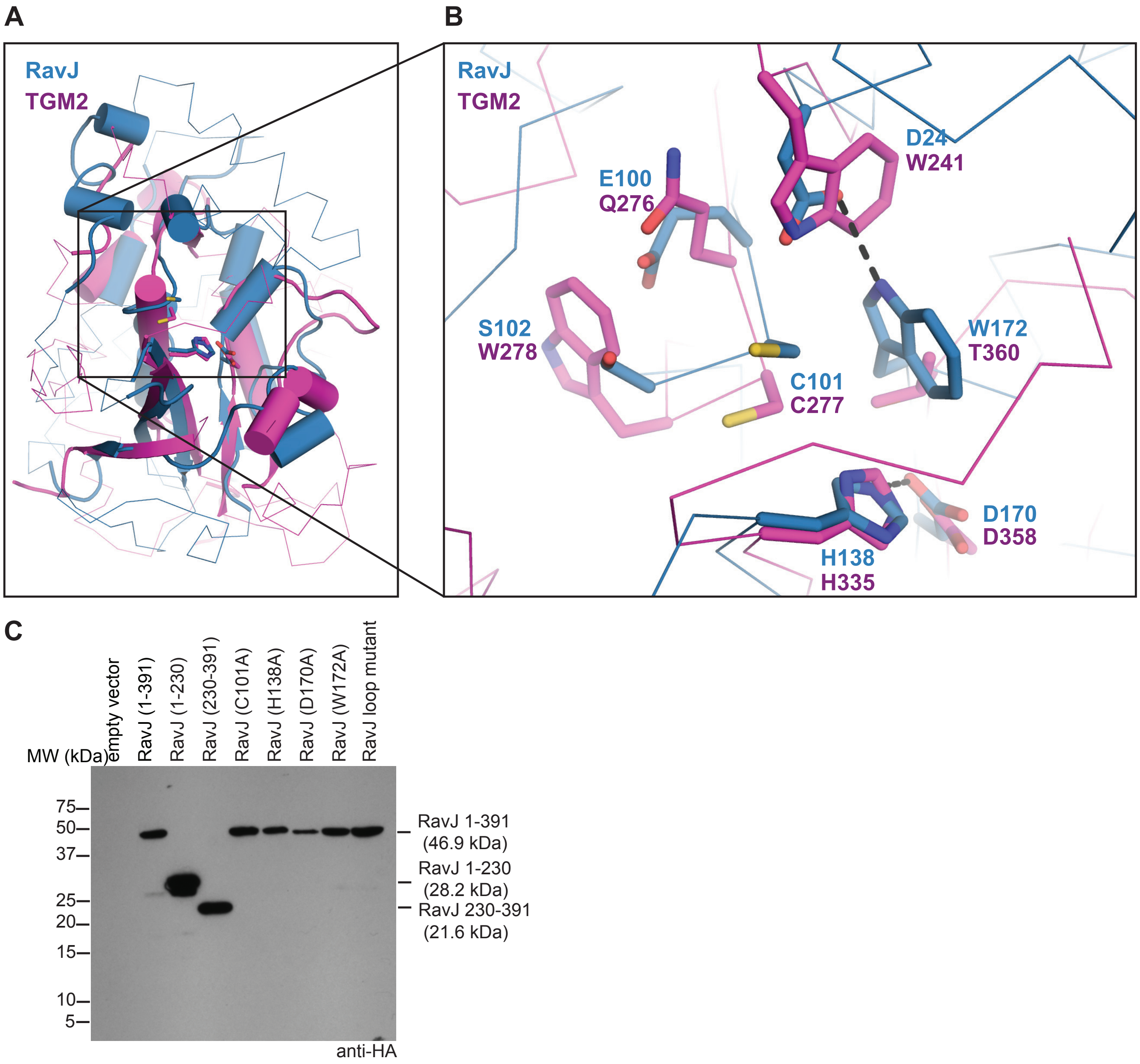

(A) The search for structural homologues for RavJ N-terminal (1-230) domain structure (blue) using the COFACTOR server at http://zhanglab.ccmb.med.umich.edu/COFACTOR/ identified the structure of human tissue transglutaminase (TGM2) (PDB: 2Q3Z)(pink) as the top structural hit (TM-score of 0.494). The superimposition of RavJ and TGM2 structures using PDBeFold, (http://www.ebi.ac.uk/msd-srv/ssm/) shows RMSD of 3.15 Å over 123 matching Cα atoms. Equivalent regions in two structures are shown in cartoon representation, with the rest of the structures shown in thin ribbon format. The side chains of catalytic triad (Cys-His-Asp) residues in both structures are shown in sticks. (**B**) Close-up view of active sites of RavJ N-terminal (1-230) domain structure superimposed with that of TGM2. The equivalent residues of the active centers in both structures including those of Cys-His-Asp catalytic triads are shown in sticks. (**C**) Expression of the RavJ mutants used to interrogate the function of RavJ in yeast spot dilution assays, are expressed and migrate approximately at their expected molecular weight (including 2.7 kDA for 1X HA and linker sequence) as indicated. Samples of cells induced for 5 hours with galactose were analyzed by SDS-PAGE and Western blot using anti-HA antibodies as described in the SI Materials and Methods.

Figure S5 - Identification of the RavJ domain interacting with LegL1; RavJ-septin complex co-immunoprecipitation.

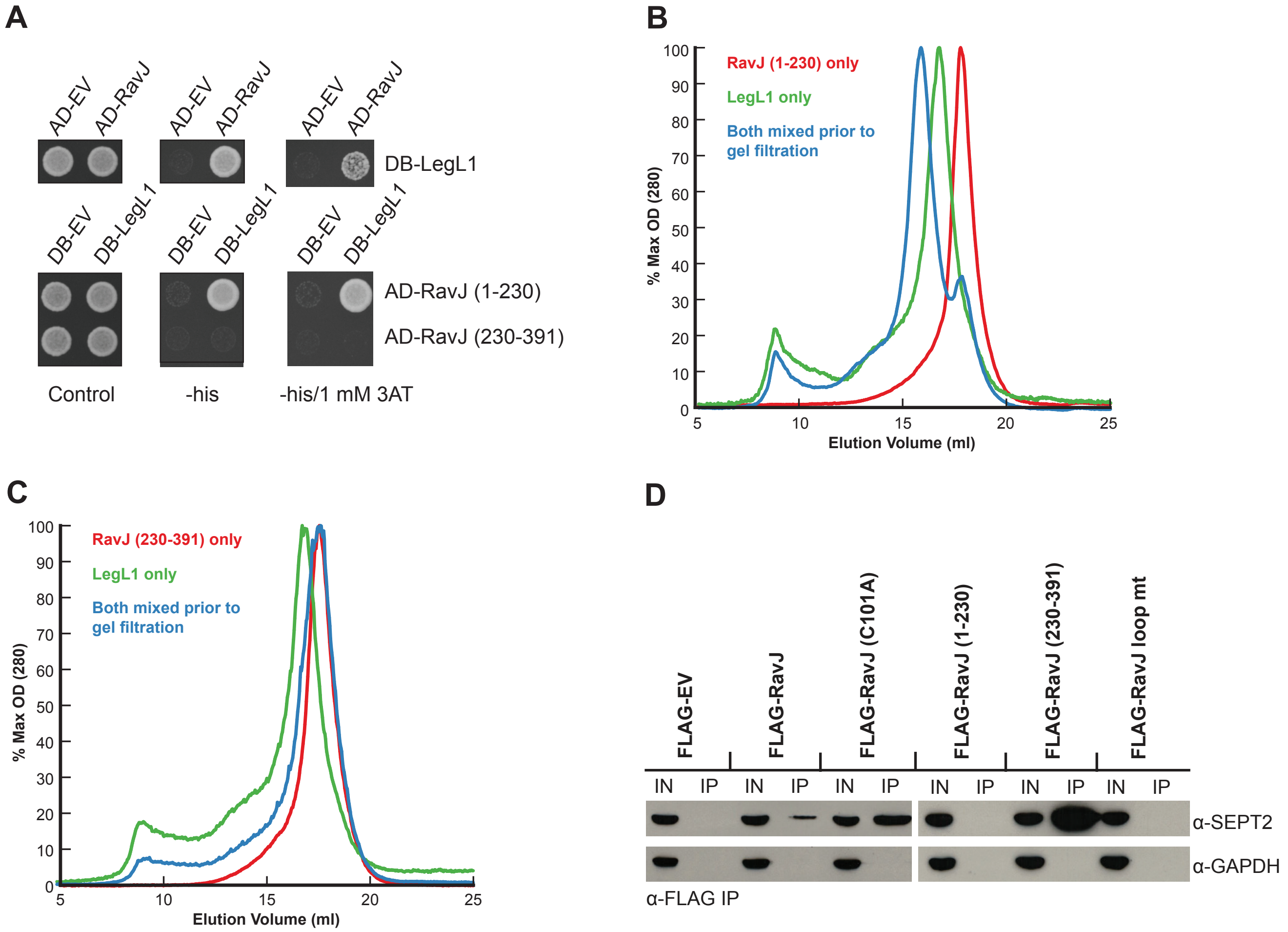

(**A**) The yeast two-hybrid assay was used to determine which domain of RavJ interacts with LegL1. Full-length and N- and C-terminal fragments (1-230 and 230-391) of RavJ were fused to the Gal4 activating domain (AD) and LegL1 to the Gal4 DNA binding domain (DB). Physical interactions between AD and DB fusion proteins drive the expression of the reporter gene HIS3, resulting in the ability to grow under –his and –his/1mM 3AT conditions. Full-length RavJ and the N-terminal domain (1-230) DB fusions with AD-LegL1 can grow on –his and –his/ 1 mM 3AT medium, while the C-terminal RavJ domain (230-391) DB fusion cannot, indicating that LegL1 interacts with the N-terminal domain of RavJ. (**B**) Purified RavJ (1-230) and full-length LegL1 were subjected to analytical gel filtration and elute as separate peaks (red and green curves, respectively). Pre-mixing of RavJ (1-230) and LegL1 prior to gel filtration resulted in a shift in the elution time consistent with complex formation (blue curve). (**C**) Purified RavJ (230-391) and full-length LegL1 were also subjected to analytical gel filtration. Pre- mixing of RavJ (230-391) and LegL1 prior to gel filtration yielded an elution profile consistent with no complex formation between the two. Due to the relatively small difference in molecular weight between these two species, monomeric LegL1 is predominantly visible as an increased signal at the front of the main peak. (**D**) RavJ's interaction with septins is confirmed by co-immunoprecipitation. HeLa cells were transiently transfected with FLAG-tagged RavJ or the constructs indicated. Western blot analysis of co-immunoprecipitations performed using anti-FLAG M2 antibodies shows that Sept2 binds to RavJ via its C-terminal domain and requires the C-terminal conserved motif.

Figure S6 - Lpg1148 (LupA) has similarities to ubiquitin or ubiquitin-like protease (UBP) family members.

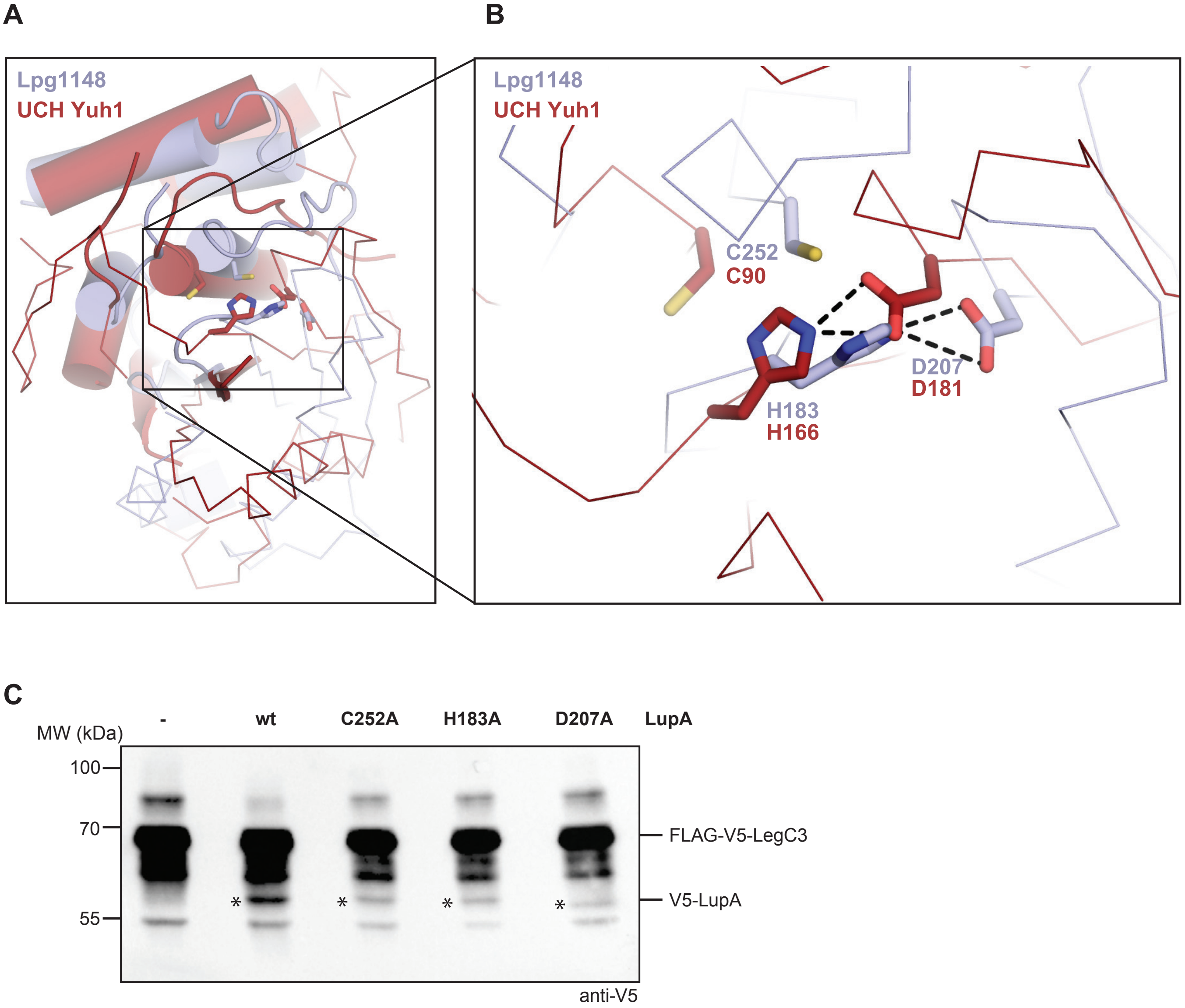

(A) The superimposition of Lpg1148 (LupA, blue) and ubiquitin C-terminal hydrolase (UCH) Yuh1 (PDB 1CMX, red) using PDBeFold, (http://www.ebi.ac.uk/msd-srv/ssm/) shows RMSD of 4.6 Å over 93 matching Cα atoms. Equivalent regions in two structures are shown in cartoon representation, with the rest of the structures shown in thin ribbon format. The side chains of catalytic triad (Cys-His-Asp) residues in both structures are shown in sticks. **(B)** Close-up view of active sites of LupA superimposed with that of Yuh1. The equivalent residues of the Cys-His-Asp catalytic triads are shown in sticks. (**C**) Wild-type and mutant LupA are expressed in the LegC3 denaturing IP. Input from a cotransfection of V5-LupA, FLAG-V5-LegC3 and HA-Ub was analyzed by SDS-PAGE and Western blot using anti-V5 antibodies. V5-LupA (*, 59.5 kDa) and FLAG-V5-LegC3 (70.6 kDa) run at their expected molecular weight and are indicated.

Figure S7 - The physical interaction between MavQ and SidP is conserved in *L. dumoffii*.

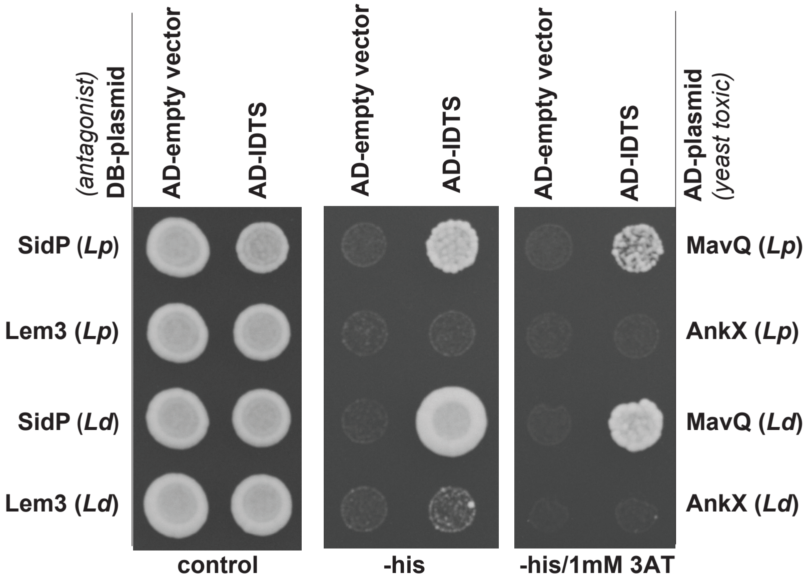

A yeast two-hybrid (Y2H) assay was performed on the antagonist pairs MavQ-SidP and AnkX-Lem3 from *L. pneumophila* (*Lp*) and their orthologs from *L. dumoffii* (*Ld*). MavQ and AnkX were fused to the gal4-activating domain (AD), and their cognate DITS SidP and Lem3 fused to the Gal4 DNA-binding domain (DB). Physical interactions between DB and AD drive the expression of the *HIS3* gene and are manifested as the ability for a strain to grow under –his and -his/1 mM 3AT conditions MavQ-SidP physically interact when derived from the same *Legionella* species. (Note that a cross-species Y2H assay is not possible between these pairs due to the failure of SidP to rescue the toxicity of MavQ from another species.)

Figure S8 - SidP and Lem14 supporting information.

A

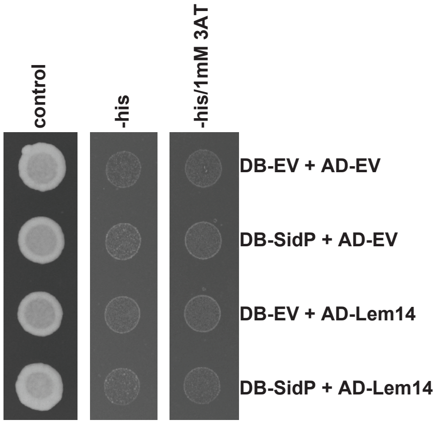

B

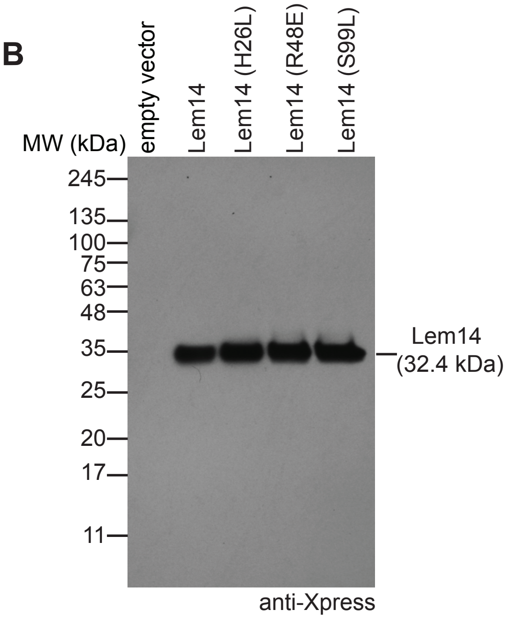

(A) Lem14 and SidP do not have a Y2H interaction. Lem14 was fused to the gal4-activating domain (AD) and SidP was fused to the Gal4 DNA-binding domain (DB). Physical interactions between DB and AD drive the expression of the *HIS3* gene and are manifested as the ability for a strain to grow under –his and -his/1 mM 3AT conditions. There is no growth on -his, indicating there is no Y2H dectectable interaction. (**B**) The Lem14 mutants used for the SidP synergy experiments are expressed and migrate approximately at their expected molecular weight (including 4.2 kDa for the Xpress tag) as indicated. Samples of cells induced for 5 hours with galactose were analyzed by SDS-PAGE and Western blot using anti-Xpress antibodies as described in the methods.

Figure S9 - Differential transcription of effectors and their antagonists.

Data from Weissenmayer, B.A., Prendergast, J.G., Lohan, A.J., and Loftus, B.J. (2011). PLoS One 6, e17570.

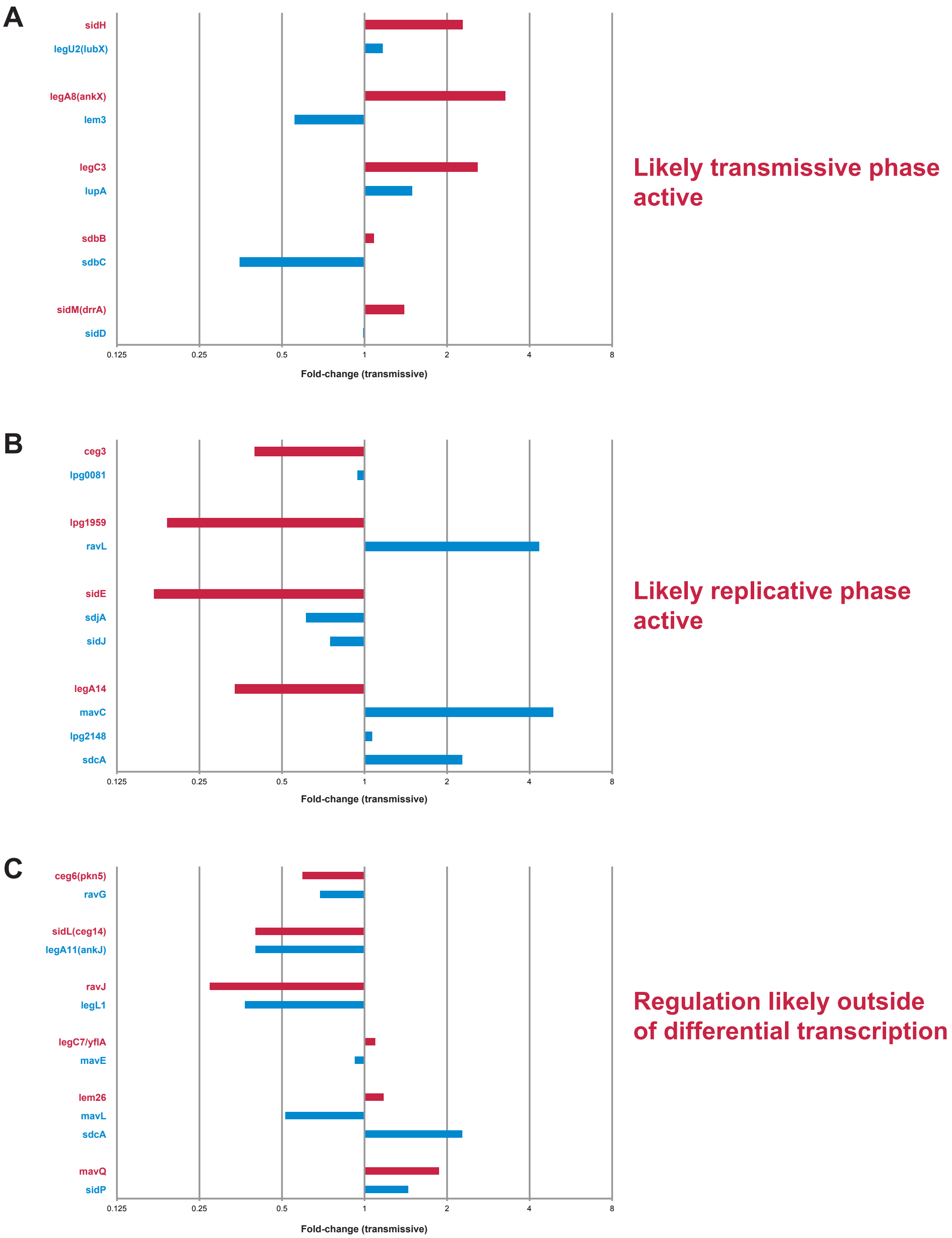

Summary of published RNA-seq data of effector transcriptional changes during intracellular replication in *Acanthamoeba castellanii*. Replicative phase was defined as 11 hours post-inoculation, transmissive phase, which generally is thought to represent very early and late stages of infection, was defined as 14 hours post-inoculation. Fold-change is the ratio of normalized reads per million (transmissive/replicative) for each gene. Cognate effectors are in red, and the (meta) effectors that regulate them are in represented in blue.
